# Women with Premenstrual Dysphoric Disorder experiences of suicidal thoughts and behaviours: a mixed methods study

**DOI:** 10.3389/fpsyt.2024.1442767

**Published:** 2024-11-06

**Authors:** Danielle Brown, Debbie M. Smith, Elizabeth Osborn, Anja Wittkowski

**Affiliations:** ^1^ Division of Psychology and Mental Health, Faculty of Biology, Medicine and Health, University of Manchester, Manchester, United Kingdom; ^2^ Psychological Services, Greater Manchester Mental Health National Health Service (NHS) Foundation Trust, Manchester, United Kingdom; ^3^ Paediatric Psychology Department, Glan Clwyd Hospital, Betsi Cadwaladr University Health Board, Rhyl, Wales, United Kingdom; ^4^ Manchester Academic Health Science Centre, Manchester, United Kingdom

**Keywords:** women’s health, mental health, women, qualitative, quantitative

## Abstract

**Background:**

Women with Premenstrual Dysphoric Disorder (PMDD) experience debilitating psychological symptoms during each luteal phase of their menstrual cycle. Although women’s increased risk of suicidal thoughts and attempts to end their lives has been highlighted, little is as yet known about women’s own narratives. Therefore, this study aimed to explore the prevalence and the experiences of self-harm, suicidal thoughts and attempts in women with PMDD.

**Method:**

In this mixed methods study, women’s experiences of living with PMDD were captured via an online survey exploring risk and via semi-structured interviews with questions informed by survey results. Data were analysed using descriptive statistics and reflexive thematic analysis.

**Results:**

Over 3,600 women completed the survey between January and March 2020 and 14 women were subsequently interviewed in 2022. The survey revealed that just under half of women had deliberately harmed themselves during a PMDD crisis, 82% had suicidal thoughts on one or more occasions during their luteal phase, and 26% of women had attempted to end their own life. Three main themes, which related to suicidal ideation in the context of PMDD, were generated from the interview data: 1) personal relationships and social connections affected by PMDD, 2) the increase in suicidal experiences caused by diagnosis delays and 3) self-worth damaged by PMDD.

**Conclusion:**

Women living with PMDD are at high risk of self-harm, suicidal thoughts and attempts to end their lives each month, narratives highlighted the damaging impacts PMDD had on relationships. Risk was increased by delays in appropriate medical support and women reported internalising their experiences as their problem.

## 1 Introduction

Premenstrual Syndrome (PMS) is cyclical and characterised by psychiatric and somatic symptoms (1, 2), peaking in the luteal phase, and resolving after the onset of menstrual bleeding (2). Approximately 50-80% of women of reproductive age have mild PMS, and 30-40% report a severity of symptoms requiring treatment (3). In 1987, the DSM-III (4) categorised a severe form of PMS, originally titled Late Luteal Phase Dysphoric Disorder and later changed to Premenstrual Dysphoric Disorder (PMDD) (5). PMDD affects 5-8% of women of reproductive age (5, 6) and is a complex, disabling condition believed to be associated with the fluctuation of hormones during the luteal phase of the menstrual cycle (7). The treatments available for PMDD are limited, often focussing on symptom reduction (1). Women living with PMDD report various cognitive, psychological and somatic symptoms; some women experience increased stress, depression, anxiety and even psychotic episodes (8–11).

Suicide is a global problem, occurring throughout the lifespan and across diverse backgrounds (12). The relationship between self-harm, suicidal ideation and suicide attempts has been explored (13), with non-suicidal self-harm being the strongest predictor of suicidal thoughts which can lead to suicide attempts (14). Statistics show that within the general population women were three times more likely to attempt suicide than men (15). In addition, a significantly higher prevalence of PMDD has been found amongst women hospitalised for attempting to end their lives (16, 17). Three large cohort publications examining correlations found significant relationships between PMDD and suicidal ideation, suicide plans and attempts (18–20). In their systematic review of ten studies, Osborn et al. (21) also noted that PMDD was associated with suicidal thoughts, plans and attempts. Furthermore, survey data indicated that women with PMDD reported higher rates of self-injurious thoughts and behaviours, when compared to population averages for women without PMDD (22). This finding was based on 599 women with a prospectively confirmed diagnosis of PMDD. However, no association between suicide attempts and different phases of the menstrual cycle for women with PMDD was identified by Osborn et al. (21), as would have been anticipated. These discrepancies in studies may reflect differences in the severity of PMDD symptoms and diagnostic methods used for its identification (21). Osborn et al. (22) noted that it could take 20 years for a women’s PMDD symptoms to be accurately diagnosed and treated, increasing the importance of further research to understand the psychological impacts that living with PMDD has on women’s experiences of suicidal thoughts and behaviours (17) both before and after diagnosis.

There is a growing literature aiming to explore the experiences of women with a premenstrual diagnosis. In order to understand the psychological impact of living with a premenstrual disorder (PMD), Brown et al. (24) conducted a metasynthesis of 17 qualitative papers and identified two themes 1) “*controlled by PMD’s*” and 2) “*a women and a life left broken*”. In their review of 12 qualitative papers, Moe and Karlsson (25) focussed on PMDD only and identified two themes: 1) “*limitations due to PMDD”* and 2) “*attempts to manage life with PMDD*”. However, Moe and Karlsson’s review (25) presented recommendations for nursing. Despite insightful findings, neither review (24, 25) captured women’s perspective of self-harm, suicidal thoughts or suicide attempts, in the context of their PMD. However, Osborn et al. (23) interviewed 17 women regarding their journey to receiving a PMDD diagnosis and identified that feeling hopeless in the context of their PMDD appeared to be linked to suicidal ideation and sometimes to women’s attempts to end their lives.

To date, no study has focussed solely on women’s experiences of self-harm and suicidal ideation in the context of their PMDD. Thus, the aims of this study were twofold: 1) to examine how many women with PMDD reported the experiences of self-harm, suicidal thoughts or attempted suicide and 2) to explore women’s lived experiences of PMDD, with a focus on their experiences of self-harm, suicidal thoughts and behaviours.

## 2 Method

### 2.1 Design and ethical approvals

A mixed methods design was chosen to explore the above-mentioned aims. Utilising quantitative and qualitative methodologies (26) enabled the development of an enhanced understanding of the impact of PMDD on women.

The survey was granted ethical approval via the British Broadcasting Corporation (BBC) internal processes, including a review by the BBC data protection team who provided a privacy notice to be presented alongside the survey. A data sharing agreement was signed between the BBC and University of Manchester. Ethical approval for the interviews was granted by the University of Manchester in March 2022 (Ref: 2022-12850-22464). Due to risk concerns and the potential need to get medical or psychological support for an individual, ethics for the interviews was only granted for the additional recruitment of women currently living in the UK.

### 2.2 Survey overview

In 2020, BBC news aired a PMDD documentary called *“My periods made me suicidal”* (27). During the production of the show a survey was completed by thousands of women internationally. The survey was designed by the BBC to capture information regarding the wider impact of PMDD on a woman’s life. The present study utilised sections of the BBC dataset.

### 2.3 Survey participant eligibility and recruitment

Participants were women aged ten years and over, who reported receiving a formal diagnosis of PMDD from a healthcare professional. As the survey was available online, it was accessible worldwide. The survey was advertised via social media accounts managed by the BBC and the *International Association for Premenstrual Disorders* (IAPMD) (28).

### 2.4 Survey procedure

Potential participants were presented with an electronic information sheet followed by 14 multiple-choice questions, with consent implied by the completion of the survey. For the purpose of the current study, only data relating to demographic information (e.g., age and years since diagnosis) and questions relating to self-harm, suicidal thoughts and attempted suicide were selected for analysis.

### 2.5 Survey data analysis

Six multiple-choice questions were selected from the larger anonymous survey dataset: three questions gathered demographic information (e.g., age and years since diagnosis) and three questions related to self-harm, suicidal thoughts, and attempted suicide. Survey responses were made available within Microsoft Excell and descriptive statistics were used, with data tabulated.

### 2.6 Interview overview

In the qualitative part of this mixed methods study, women were interviewed to collect lived experience data, which were analysed using reflexive thematic analysis (29). This method allows for further exploration of participant experiences and perspectives, beyond the quantitative data available from the survey. The survey findings informed the development of the qualitative topic guide used in the subsequent interviews with British women.

### 2.7 Interview participant eligibility and recruitment

All participants were English speaking women, over 18 years old, living in the UK, who reported receiving a formal diagnosis of PMDD, their symptoms were tracked for a minimum of two months by their medical doctor. Participants were recruited via a social media advert shared online and within premenstrual disorders forum pages between April and September 2022. Potential participants, who contacted the research team to express interest, were emailed a copy of the information sheet and consent form.

### 2.8 Interview development and data collection

Although the survey captured the data from a large number of women with PMDD, it included pre-defined multiple-choice questions. To understand the wider ranging impact of PMDD on women, a topic guide was developed based on the survey results, which included questions and prompts related to experiences of self-harm, suicidal thoughts and suicide attempts. Additionally, a demographic questionnaire was developed to record their use of symptom trackers, estimated age of symptom onset and age of diagnosis.

### 2.9 Interview procedure

Interviews were completed via video call, once the participant had provided written informed consent and completed the short demographics questionnaire. A semi-structured topic guide was followed, and interviews were recorded, securely stored and transcribed verbatim.

As participants were not recruited through a medical team, all participants provided their GP details and consented to them being contacted, in case there were any risk or safeguarding concerns raised during the interviews. All participants were made aware they had two weeks after the interview date to withdraw, after this time all data would be anonymised and could not be identified. Due to the emotive topic, a distress management plan and debrief sheet were developed to protect their wellbeing.

### 2.10 Interview data analysis

The analysis of the qualitative data was guided by the six stages of Braun and Clark’s reflexive thematic analysis (29, 30). This method was chosen to understand the participants’ experiences with theoretical freedom.

Two authors (DB and DMS) familiarised themselves with the interview dataset, taking an inductive approach to code line-by-line and separately generate initial semantic codes, before jointly reviewing preliminary latent codes (phase 2). Both NVivo and paper post-it notes were used to collate comparable codes and construct themes to conceptualise patterns of shared meaning shown by the participants (phase 3). Once latent themes were described, further analysis of the dataset was undertaken (by DB and DMS) to ensure the themes represented and reflected the experiences of the participants (phase 4). Themes were then defined and named (phase 5) through consultation with the research team, before the written report was produced (phase 6).

### 2.11 Reflexivity and rigour

The first author (DB) was a Trainee Clinical Psychologist, with a history of working with children, families and working in complex, challenging environments, such as secure services. The second author (DMS) was a Health Psychologist and Senior Lecturer, with a background in exploring pregnancy and behaviour change. The third author (EO) was a Clinical Psychologist working within paediatric services and had a specialist research interest in premenstrual disorders. The fourth author (AW) was a Clinical Psychologist and Senior Lecturer, whose clinical work involved supporting mothers with severe mental health difficulties therapeutically. The authors had varying levels of qualitative research experience and of clinical support for women with premenstrual conditions. As all authors were women and mothers, these roles could have impacted how they viewed the data, given all participants were also women and many, but not all, were mothers. To reduce bias during analysis, the first author kept a reflective journal, which she used to write down thoughts and impressions during interviews, transcription, and coding, as well as methodological decisions. These reflections were shared with all authors, when appropriate, and discussed during coding and theme generation, allowing a secondary level of reflection and consideration. The variety of experience and clinical backgrounds allowed for authors to challenge each other’s assumptions and pre-conceptions.

## 3 Results

### 3.1 Survey participant characteristics

Overall, 3,906 women with a reported diagnosis of PMDD from a healthcare professional completed the survey between January and March 2020. The age of participants ranged from ten to over 60 years old and the majority were non-UK residents. Whilst most women received their diagnosis in the last nine years, some women reported receiving a diagnosis over 30 years ago (see **Table 1** for further details).

**Table 1:** Demographic characteristics for survey data.

		N (%)
Country	UK Resident Non-UK Resident Unknown	1,547 (40%) 1,979 (51%) 380 (9%)
Age	Under 19 20 – 29 30 – 39 40 – 49 50 – 59 Over 60 Unknown	85 (2%) 970 (25%) 1,733 (44%) 1,002 (26%) 109 (3%) 2 (<1%) 5 (<1%)
Years since diagnosis	0 – 9 years 10 – 19 years 20 – 29 years Over 30 years Unknown	1,825 (47%) 1,213 (31%) 663 (17%) 143 (4%) 62 (2%)

### 3.2 Survey findings

Of 3661 women, 47% reported to have self-harmed, at least once during a PMDD crisis (see **Table 2**). Specifically, 9% regularly self-harmed, whilst 31% had self-harmed on occasion and 7% reported only one instance of self-harm related to their PMDD. The remaining women had self-harmed at a different time in their menstrual cycle (3%) or had never self-harmed (49%).

**Table 2:** To what extent had PMDD led to self-harm?

1. To what extent had PMDD led to self-harm?	Number (%)
I regularly self-harm during PMDD crises	322 (9%)
I have self-harmed on occasion during PMDD crises	1150 (31%)
I have self-harmed once during a PMDD crisis	274 (7%)
I have self-harmed but at a different time/s in my menstrual cycle	116 (3%)
No, I have never self-harmed basis just before my period	1799 (49%)
	3661

In total, 3768 women responded to question 2; 82% had experienced suicidal thoughts, on one or more occasion during their luteal phase (see **Table 3**). Of these, 6% of women reported suicidal thoughts once, and 36% had experienced suicidal thoughts on occasion. Forty percent of respondents experienced suicidal thoughts monthly.

**Table 3:** To what extent had PMDD led to suicidal thoughts?

2. To what extent had PMDD led to suicidal thoughts?	Number (%)
I have suicidal thoughts on a monthly basis just before my period	1511 (40%)
I have suicidal thoughts on occasion just before my period	1364 (36%)
I have had suicidal thoughts once just before my period	244 (6%)
I have had suicidal thoughts but at a different time/s during my menstrual cycle	286 (8%)
No, I have never had suicidal thoughts	363 (10%)
	3768

Of 3670 women, 13% reported to have attempted suicide more than once and 13% had attempted suicide once during a PMDD crisis (see **Table 4**). Two percent of women responding had attempted suicide at different times in their menstrual cycle, unrelated to their PMDD and 72% had never attempted suicide.

**Table 4:** To what extent had PMDD led to attempted suicide?

3. To what extent had PMDD led to attempted suicide?	Number (%)
I have attempted suicide more than once during PMDD crisis	479 (13%)
I have attempted suicide once during a PMDD crisis	477 (13%)
I have attempted suicide but at a different time/s in my menstrual cycle	69 (2%)
No, I have not attempted suicide	2645 (72%)
	3670

### 3.3 Interview participant characteristics

Thirty-three women contacted the researcher, three women were not living in the UK and were therefore unable to participate, in line with ethical approval. Fifteen individuals did not respond to follow up emails. Thus, 14 women consented to take part and were interviewed between May and September 2022. Their ages ranged from 24 to 54 years, and they were all White British. Although the mean age of symptom onset was 22 years old, the mean age of diagnosis was not until 42 years old. All 14 women had received specialist treatment previously, eight were biological mothers of children ranging from three to 26 years old, one woman had stepchildren (see **Table 5** for further demographics).

**Table 5:** Demographic characteristics of interviewed participants.

		N (%)
Age	Under 19 20 – 29 30 – 39 40 – 49 50 – 59 Over 60	0 (0%) 3 (21%) 2 (14%) 7 (50%) 2 (14%) 0 (0%)
Ethnicity	White	14 (100%)
Highest level of education	No school attended Primary school Secondary school Collage University Other…	0 (0%) 0 (0%) 2 (14%) 3 (22%) 9 (64%) 0 (0%)
Marital Status	Single In a relationship Co-habiting Married Divorced Other…	3 (21%) 0 (0%) 5 (36%) 5 (36%) 1 (7%) 0 (0%)
Estimated age of symptom onset	Under 19 20 – 29 30 – 39 Over 40	11 (79%) 2 (14%) 1 (7%) 0 (0%)
Age when diagnosed	Under 19 20 – 29 30 – 39 40 – 49 Over 50	0 (0%) 5 (36%) 3 (21%) 7 (50%) 0 (0%)
Currently receiving treatment	Yes No	12 (86%) 2 (14%)
Previously received treatment	Yes No	14 (100%) 0 (0%)
Have children	Yes No	9 (64%) 5 (36%)

### 3.4 Thematic analysis themes

**Figure 1:**
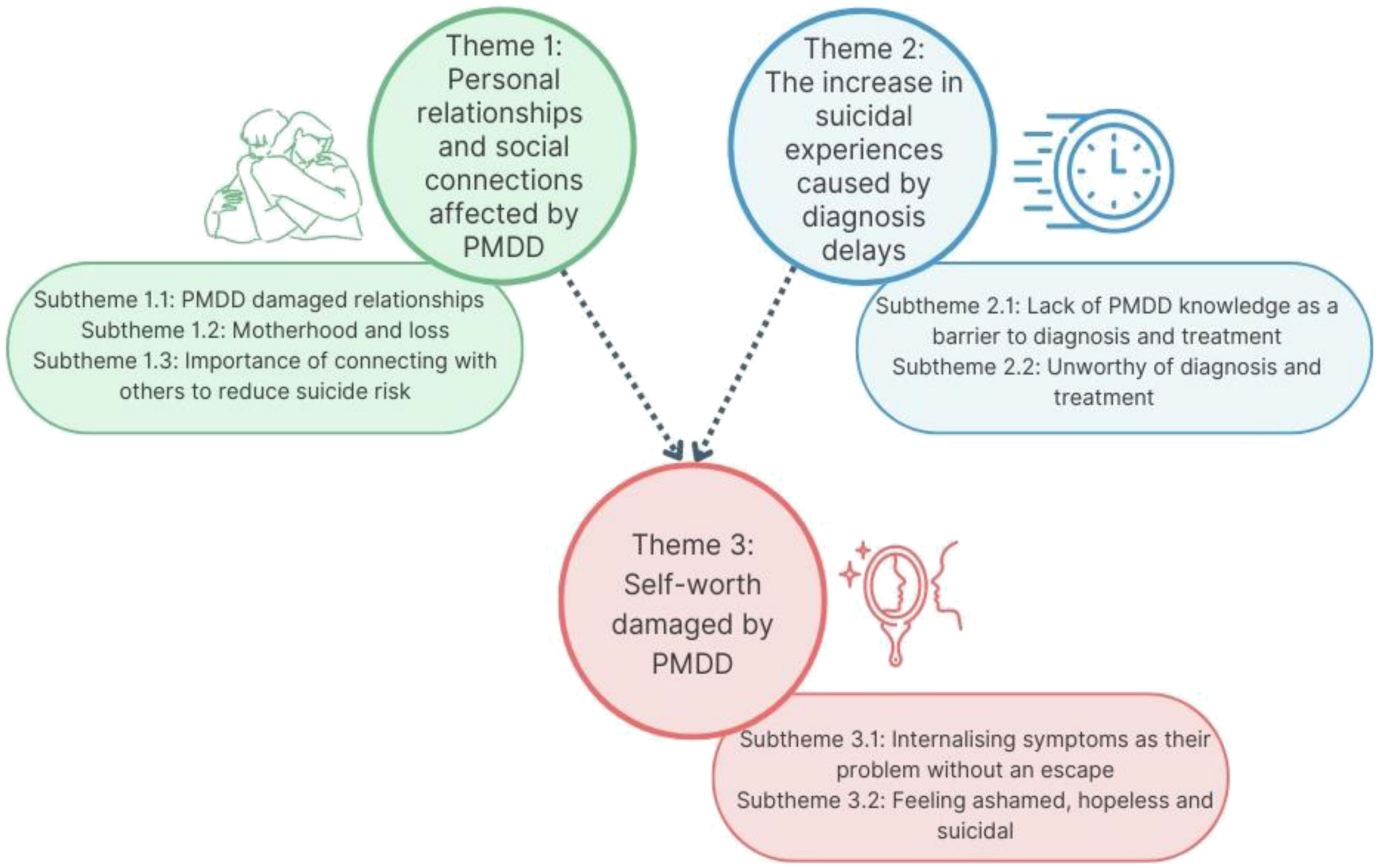
Diagram of themes from interviews.

Three themes were developed from women’s narratives that reflected the psychological impact PMDD had on them, including their experiences of suicidal ideation and attempt (see **Figure 1** for a conceptual diagram). The experiences captured within themes one and two had a direct impact on women’s self-worth, as described in theme three. The three themes will be presented below with related subthemes (n=7) and pseudonymised quotes from the transcripts.

#### 3.4.1 Theme 1: Personal relationships and social connections affected by PMDD

Women’s narratives revealed numerous attempts of engaging in personal relationships and social connections whilst living with PMDD and associated suicidal thoughts each month or during a crisis. These experiences were described as complex and at times chaotic. Women recognised their relationships being negatively impacted or damaged, contributing to emotional isolation, and this in turn contributed to their experiences of suicidal thoughts or attempts. Women spoke about their romantic relationships, family members and friendships; however, they particularly highlighted the greater impact PMDD had on their role as a mother, or their ability to become a mother. This main theme consisted of three subthemes.

##### 3.4.1.1 Subtheme 1.1: PMDD damaged relationships

In all cases, woman described a perceived link between their experiences of suicidal thoughts and the key relationships with other adults in their lives. A common view amongst the women was a recognition that these relationships were reciprocal for approximately three weeks each month. But when in a PMDD crisis and experiencing associated suicidal thoughts, this dynamic changed. One woman described the difference in her interacting with her loved ones during each luteal phase as “*unreasonable*” (Sally). Women spoke of ending their romantic relationships monthly, long-term friendships being broken and struggles to maintain connections with colleagues, all of which left them hopeless.

*“Demons just came out and suddenly that friendship was never the same” (Catherine).*

Relationships and friendships were categorised by push-pull patterns, with women trying desperately to repair the damage caused by their suicidal thoughts linked to their PMDD crisis, whilst knowing they did not have long until the same pattern repeated itself. Upon recognition of this pattern, women reported increased feelings of hopelessness and subsequent suicidal thoughts or attempts. Women described that living with them was like living with a “*roller coaster*” (Katie), and that they noticed psychological distress being experienced by their partners. According to women, partners often particularly struggled to understand the unpredictable changes and suicidal experiences expressed by them.

*“He used to say I just didn’t know what I was going to come home to. Said you’d be fine, I’d walk out the room, and I’d come back in and your face would have changed and you’d be raging about nothing and everything. Erm and at my worse he didn’t know if he was going to come home and find me having killed myself”* (Katie).

##### 3.4.1.2 Subtheme 1.2: Motherhood and loss

A women’s role as a mother was described with more importance, different dynamics, and expectations to their other relationships, as detailed in the previous subtheme. Motherhood was an emotive topic discussed by all the women and was often underpinned with feelings of loss. Whilst some women described their monthly suicidal experiences as robbing them of the opportunity to be a mother, those who had children recognised the loss of the mother they had wished to be.

Furthermore, worrying about the impact PMDD would have on their children was a common experience, and some women made the difficult decision not to have children, instead choosing to have a hysterectomy and, hopefully, live a functional life. There was recognition that not being able to have children impacted on their current or future partners.

*“I chose not to have kids because it was so bad and I thought I really don’t want to, you know, I really don’t want to hurt, and it’s awful because I love kids”* (Hannah).

Another concern women spoke of was how they would be able to prioritise taking care of a child, when they did not feel able to care for themselves for a period of time each month. Many women also expressed worries about the psychological impact PMDD would have on their future children.

*“I was really worried about like emotional blueprint and transference, and what happens and how traumatic potentially the child’s first twelve, twenty-four months could be, ‘cos even if I had the child, then what happened about the attachment and things like that, and I was like, it’s one thing having, needing like psychiatric care and ending up in a mother and baby unit potentially, when you don’t know, but if you anticipate that is what’s going to happen, and you think that’s likely, I was like I don’t, I don’t think I can do that to a child”* (Natalie).

Experiencing suicidal thoughts whilst parenting left women with indescribable guilt, and worry for the impact this could, or did have, on their children. Some women described what their children witnessed as trauma and worried about the possibility of their daughters also being diagnosed with PMDD.

*“Erm, it’s then it’s the guilt, pressure of you know you feel low and you’re shutting down on people, but you can’t help it and being a mum, you’ve got the mum pressure of, you know, needing to be there for your children and not wanting them to see you in that kind of frame of mind, but you can’t do anything about it, it just completely takes over”* (Elisa).

##### 3.4.1.3 Subtheme 1.3: Importance of connecting with others to reduce suicide risk

Women’s narratives offered ideas on what might help them cope during a mental health crisis related to their PMDD. They emphasised the importance and value of being able to connect with at least one person. Women valued this connection and viewed it to reduce the risk of acting on their impulses to self-harm or attempt to end their lives.

*“I’m feeling really low, like I just feel like I could just literally just end it, erm, and she just stayed with me and just talked to me and just listened and that kind of got me through the crisis point”* (Ellie).

Unfortunately, women’s suicidal thoughts were described as creating an emotional barrier, preventing women from connecting with other people the way they wished to.

*“Because of how you are and what you’re struggling with, so that, I think makes you feel even more lower, and more likely to feel suicidal because you just think, everyone’d be so much better off without me, because nobody understands what you’re going through”* (Ellie)

Women described feeling emotionally isolated in their experiences: their friends and family rarely understood their diagnosis of PMDD, their suicidal thoughts or why their behaviour would change so drastically each month. Feeling isolated meant that women did not disclose their suicidal thoughts, perpetuating the experience of isolation. Some women described comparing their premenstrual symptoms to those of their friends, the recognition of differences left women feeling “*alone*” (Catherine).

*“Nobody feels like this, nobody feels the way I do, nobody knows how I feel”* (Antonia).

#### 3.4.2 Theme 2: The increase in suicidal experiences caused by diagnosis delays

The process of seeking a diagnosis and treatment for their PMDD symptoms was prolonged due to repeated visits to different medical professionals, who were often unaware of PMDD. Such difficult medical appointments left women feeling both dismissed and unworthy of help because they left no closer to a diagnosis or treatment plan than before they sought help. The length of delay also drastically increased perceptions of risk for women; the longer women waited, the more menstrual cycles and suicidal thoughts they had and the number of healthcare appointments increased. Two subthemes supported this main theme.

##### 3.4.2.1 Subtheme 2.1: Lack of PMDD knowledge as a barrier to diagnosis and treatment

Delays in receiving a diagnosis and suitable treatment plan prolonged the hopelessness women reported feeling, which women linked this to an increased risk of suicidal thoughts. They described their journey to diagnosis and treatment as characterised by repeated visits and referrals to a wide variety of services, most of which had minimal knowledge or awareness of PMDD.

*“Sort of reflecting on it all, I think, with that lack of support, without that understanding, etc, it put me so, so much lower, and more at risk of self-harm and suicide”* (Ellie).

Women described having had appointments with multiple different services regarding their undiagnosed PMDD, including, general practice doctors, community mental health teams, outpatient crisis teams, personality disorder services and some were inpatients on acute mental health wards. Each referral returned the women back to an indefinite wait for support they perceived a diagnosis or treatment would bring, whilst living with significant risk of self-harm or suicidal behaviours each menstrual cycle. *“PMDD just, kind of as a tsunami just washing over me, just month after month”* (Stephanie). These referrals also required women to voice their thoughts and plans to harm themselves, which was reported as incredibly traumatic, and was only made worse by the lack of appropriate support which often followed.

*“Everything takes such a long time, doesn’t it, so you’re waiting, waiting, waiting, which makes everything worse, and it makes, probably makes you more angry and more tense and more anxious and depressed, because you’re currently waiting and waiting”* (Scarlett).

##### 3.4.2.2 Subtheme 2.2: Unworthy of diagnosis and treatment

When seeking support for their PMDD symptoms, including thoughts to harm themselves, from healthcare professionals, women described feeling dismissed. They recounted stories of being called a “*hypochondriac*” (Lauren), “*told to go away*” (Sally) and left appointments feeling “*pathetic*” (Catherine) that they were not able to manage their symptoms. Interactions with healthcare professionals left women feeling their experiences did not deserve a diagnosis and they were not worthy of the clarity women hoped a diagnosis could provide.

*“She didn’t know what to do, she found my suicide letter phoned the ambulance, and when I was down there a nurse said what is she doing here, she was safe in her house, she’s wasting our time, so they then left me for 12 hours of my own, whilst waiting for the community mental health to come out and assess me”* (Katie).

Many spoke about being misdiagnosed: “*bipolar*” (Hannah), “*chronic fatigue*” (Sally), “*borderline personality disorder*” (Mandy) or “*eating disorder*” (Natalie). A misdiagnosis was also given despite women bringing symptom trackers, which evidenced the cyclical nature of their symptoms, to appointments. Receiving a misdiagnosis left women feeling invalidated and frustrated. Women expressed how their thoughts to harm themselves or end their lives would increase following those appointments. Women reported perceiving that they knew more about PMDD than their doctor did.

*“I would go into the doctor surgeries and be like look I’ve done some research now I’ve been coming to you on and off every month for the last few years, with the same symptoms, I think I have PMDD and they would just be like oh what’s that, I’d be like what you’re not, you don’t know what PMDD is, it would just be so debilitating because then I would have to explain my own possible condition to people that know a great deal more than me”* (Catherine).

During conversations about the treatment options for PMDD, women also reported medical doctors included the cost as one of the most important factors to consider. Often the cost was prioritised above the clinical indication, the women’s own wishes or their desire to have children. Other women reported their only option to access treatment was to seek private medical treatment.

*“I’d sell anything it’s not about money. What price are you going to pay for your life”* (Hannah).

#### 3.4.3 Theme 3: Self-worth damaged by PMDD

The risk of harm to themselves was ever present in women’s accounts of their personal relationships and interactions with healthcare professionals, as evidenced by the previous two themes. The culmination of these experiences, for most women over many years, was described as damaging to their self-worth. Women framed their symptoms as their problem and that they were flawed due to the emergence of suicidal thoughts on a monthly basis, subsequently women reported to experience extreme guilt and shame. Being overwhelmed by these emotions increased the sense of hopelessness, risk of self-harm, suicidal thoughts, and attempts. The suicidal experiences of women varied, with some reporting one past experience of suicidal ideation, and others describing monthly attempts to end their life. This final theme was supported by two subthemes.

##### 3.4.3.1 Subtheme 3.1: Internalising symptoms as their problem without an escape

Women stated that after years of damaged relationships and difficult interactions with healthcare professionals, they had internalised their experiences as their “*problem*” (Molly) or their “*fault*” (Stephanie). Others questioned the sudden desire to harm themselves each month: “*I was like, why am I like this, I don’t understand*” (Sally). Some women described that they were failing at simply being a woman, and they were worthless.

*“It was like, great, another thing that I’m shit at, basically”* (Laurie).

The perceived association between internalising symptoms and increase in risk of self-harm was expressed by women. The risk was framed as something they had to manage themselves at home, with minimal professional help. Internalising their PMDD experiences was described by women to lower their self-worth and damage how they perceived themselves.

*“I wasn’t good enough, and, you know, my inner voice was just so negative”* (Mandy).

For a smaller proportion of women, this internalising was somewhat alleviated after receiving a diagnosis, and helped women to recognise they were not to blame.

*“PMDD has made me feel like I’m such an awful person, erm along with getting used to masking putting faces on so people didn’t really know the pain and it was kind of a relief erm to actually find out that I wasn’t just vile and there was something out my control erm a reason for it”* (Katie).

##### 3.4.3.2 Subtheme 3.2: Feeling ashamed, hopeless and suicidal

As a result of internalising their experiences, women spoke of an intense “guilt” (Elisa) and shame, specifically for thoughts of self-harm or suicide during their luteal phase and for acting on these impulses. At times this shame was so intense it would stop them seeking support.

*“I have quite big scars on my arms, from where I hadn’t gone to hospital, I hadn’t had stitches ‘cos I feel so ashamed of what I’ve done”* (Stephanie).

Women described the distress and upset their self-harm caused their family and friends, and subsequently feeling “ashamed” (Mandy). Additionally, once their menstrual cycle started and their symptoms reduced, women described almost immediately feeling ashamed about their behaviours, which, unfortunately, often led to a perceived increased risk of self-harm, suicidal thoughts and attempts to end their lives.

*“You also feel like a burden as well, to everybody, because of how you are and what you’re struggling with, so that, I think makes you feel even more lower, and more likely to feel suicidal because you just think, everyone’d be so much better off without me, because nobody understands what you’re going through”* (Ellie).

## 4 Discussion

This study was the first to examine how many women with PMDD reported experiences of self-harm, suicidal thoughts and attempts, whilst also capturing women’s lived experiences, associated with their PMDD. Over 3,600 women with PMDD responded to the online survey; just under half of the women had deliberately harmed themselves during a PMDD crisis, 82% had suicidal thoughts on one or more occasion during their luteal phase, and 26% of women had attempted to end their own life. These results support previously reported rates in the literature for self-harm (51%) (22), suicidal thoughts (72%) (22) and suicide attempts (14%) (18) (30%) (22). Differences in rates reported may be linked to notably differing sample sizes (59 in (18); 599 in (22)) or variations in terminology used (e.g., suicidal thoughts versus active suicidal ideation). Therefore, authors should be clear and specific with their choice of terminology. Additionally, cultural differences may be affecting the rates reported, because our survey was open to women worldwide; however, Hong et al. (18) included women living in Korea only.

The potential link between the experiences of PMDD and self-harm, suicidal thoughts and attempts was examined in more detail through interviews. Women outlined that relationships were disrupted on a regular basis because of their PMDD, its associated symptoms and deterioration in their mental health. Our finding that medical delays and lack of PMDD knowledge within the medical profession, was described as having significant negative impacts on their self-worth and thoughts of ending their lives. These findings complement those by Osborn et al. (23) and Chan et al. (31), who explored the journey to diagnosis within the UK and US healthcare systems, respectively. Both sets of authors highlight the detrimental impact of time delays, with Osborn et al. (23) reporting an average delay of 20 years before PMDD was diagnosed. Delays in receiving diagnosis and treatment were linked to feeling unworthy and hopeless within these findings, the internalising of PMDD symptoms was perceived as fundamental to women’s risk of self-harm, suicidal thoughts and attempts to end their lives. Interestingly, specific focus was placed on motherhood and women reported markedly different dynamics in other relationships related to their PMDD.

The role of social isolation and loneliness in increasing the risk of suicide and in contributing to poorer health outcomes has been reported (e.g., mental health issues, heart attacks, stroke) (32). However, novel insights were presented as women struggled to connect with people who rarely understood their PMDD experiences, their perceived emotional isolation intensified, and they expressed thoughts of self-harm and suicide. NICE guidance for preventing suicide and managing self-harm, and for the diagnosis and treatment of PMS, including PMDD, are reported on separately (33–35). Recommendations include suicide prevention plans, family involvement in treatment planning, and multi-agency working. However, NICE guidance fails to account for women with PMDD, whose self-harm and suicidal thoughts are nuanced and cyclical. Additionally, the benefits of safety planning interventions for individuals presenting at emergency departments expressing “suicide-related concerns” [(36) p.895] have been documented. Future research and guidance specifically supporting the cyclical risk associated with PMDD should be explored.

### 4.1 Clinical implications and wider recommendations

The perceived increased risk associated with delayed access to medical support highlights the clear priority to ensure women are diagnosed in a timely fashion, and therefore receive treatment and mental health support during periods of crisis. Initiatives for all individuals presenting with risk of suicide within the Nation Health Service (NHS) have been recommended (37). However, as these do not recognise the nuances of PMDD. Routine screening for PMDD and additional training regarding PMDD awareness and signposting should be implemented within services supporting women in crisis, such as paramedics, accident and emergency, mental health services, general practice doctors.

Current findings emphasise the importance of personal relationships, both for general support and at times of crisis. Following diagnosis, healthcare services should consider ways to support women’s families, for example, via information leaflets. Additionally, as social connection is a protective factor against risk to self (32), services may consider post-diagnostic support groups, creating a space for mutual understanding and sharing experiences.

The relationship between shame and increased risk of suicidal behaviours has been described within this study and wider literature; services diagnosing PMDD should consider referrals to psychological services. Cognitive Behavioural Therapy (CBT) is a recommended modality for women with PMDD (38), and in addition Acceptance and Commitment Therapy (ACT) (39) or Compassion Focused Therapy (CFT) (40) may be helpful in addressing the risk and/or the shame experienced by women.

Education regarding menstruation is included in the UK school curriculum; however, premenstrual disorders, such as PMDD, are not specified. Many women in our study (79%) estimated their symptoms started whilst they were school age; however, 71% were not diagnosed until they were over 30 years old. If girls were educated about PMDD in school, it is possible that those who develop symptoms would recognise their experiences sooner and seek medical advice. Additionally, inclusion of PMDD within the school curriculum would increase awareness and understanding within the general population.

### 4.2 Strengths, limitations and suggestions for future studies

A strength of this study was the mixed methods approach, in which findings from the survey data were followed up by in-depth interviews. A mixed methods design enabled the aims to be studied from different perspectives: the strengths, and weaknesses of each approach complemented the other (41).

The online survey was open to women worldwide, girls and women of different ages could take part. However, it should be acknowledged that the survey questions were most likely designed with adult women in mind only. In addition, diagnosis of PMDD could not be verified for survey participants. This limitation was minimised in the interview study in which the researcher verbally confirmed diagnosis and the use of symptom trackers with each woman as part of their diagnostic assessment. Alternatively, prior to interviews a screening tool could have been used, such as the PSST (42).

Although the samples of women for the survey and the interviews were diverse in terms of age and years since diagnosis, all the interviewed women were White British and most (86%) had accessed higher education, leading to a possible bias in that study. There is also a possibility of selection bias because both the survey and interviews required internet access to participate, thereby limiting recruitment to those with computer literacy skills and those who had an awareness of social media as a means recruitment (43).

As described within the methodology, the research team consisted of women only who had varying degrees of professional and/or personal exposure to PMDD creating parallels with the participants. However, the authors remained outsiders, researchers who were studying a group of which they were not a member (44).

## 5 Conclusions

Women’s experiences of self-harm, suicidal thoughts and attempts in the context of their PMDD were explored in this mixed methods study. Survey data drawn from over 3,600 responses demonstrated prevalence rates as high as 82% for suicidal thoughts, whilst women’s narratives further illustrated the role of that emotional isolation, delayed diagnosis and feeling unworthy, had on women’s suicidal experiences. These findings underscore the need for more timely diagnosis of PMDD, better public awareness of the condition and improved health service support for women once diagnosed.

## Data Availability

The raw data supporting the conclusions of this article will be made available by the authors, without undue reservation.
